# Evaluation of PhenoMATRIX and PhenoMATRIX PLUS for the screening of MRSA from nasal and inguinal/perineal swabs using chromogenic media

**DOI:** 10.1128/jcm.01152-23

**Published:** 2023-12-21

**Authors:** Abdessalam Cherkaoui, Gesuele Renzi, Jacques Schrenzel

**Affiliations:** 1 Bacteriology Laboratory, Division of Laboratory Medicine, Department of Diagnostics, Geneva University Hospitals, Geneva, Switzerland; 2 Division of Infectious Diseases, Department of Medicine, Genomic Research Laboratory, Geneva University Hospitals and Faculty of Medicine, Geneva, Switzerland; NorthShore University HealthSystem, Evanston, Illinois, USA

**Keywords:** MRSA, chromogenic media, WASPLab, PhenoMATRIX, PhenoMATRIX PLUS

## Abstract

The objective of this study was to assess the clinical performances of PhenoMATRIX and PhenoMATRIX PLUS for the screening of methicillin-resistant *Staphylococcus aureus* (MRSA) from nasal and inguinal/perineal ESwabs using chromogenic media. The automated performances were compared to the manual reading. Additionally, we evaluated PhenoMATRIX PLUS for the automatic release of the negative results to the Laboratory Information System (LIS) and the automatic discharge of the negative plates from the incubators. A total of 6,771 non-duplicate specimens were used by PhenoMATRIX as a machine learning model. The validation of these settings was performed on 17,223 non-duplicate specimens. The MRSA positivity rate was 5% (866/17,223). Validated settings were then used by PhenoMATRIX PLUS on another 1,409 non-duplicate specimens. The sensitivities of PhenoMATRIX and PhenoMATRIX PLUS were 99.8% [95% confidence interval (CI), 99.2%–99.9%] and 100% (95% CI, 92.1%–100%), respectively. The specificities of PhenoMATRIX and PhenoMATRIX PLUS were 99.1% (95% CI, 99.0%–99.2%) and 95.2% (95% CI, 93.8%–96.1%), respectively. All the 1,297 MRSA-negative specimens analyzed by PhenoMATRIX PLUS were automatically released and sent to the LIS immediately after availability of the culture image on the WASPLab (100% accuracy). All negative media plates were automatically discarded. PhenoMATRIX PLUS decreases the time spent by technologists on negative plates and ensures optimal usage of the incubators' capacity.

## INTRODUCTION

Methicillin-resistant *Staphylococcus aureus* (MRSA) has been recognized as one of the leading causes of hospital-acquired infections and contributes to a greater increase in intra-hospital mortality, morbidity, and length of stay (1, 2). It is also being considered as an important pathogen in community-associated infections (3). The timely identification of MRSA carriers represents one of the major challenges for the infection control team (4). Appropriate measures require accurate results and rapid turnaround times (TAT). Using total laboratory automation (TLA), the MRSA culture images are available 7/7 and 24/24 independently of the operating hours of the laboratory. In routine, all culture images are read by technologists to determine the next steps for the analysis [growth or no growth, identification of the colonies required or not, and antimicrobial susceptibility testing (AST) required or not].

Diagnostic microbiology laboratories are facing several challenges but most of them, like the steady increase in the number of clinical specimens and the high diversity and complexity of procedures, have been—at least partly—addressed using TLA (5 – 10). However, manual reading of the culture images remains time consuming and extends the time to results, especially for negative media plates. This is obviously even more of concern for analyses with low positivity rates.

Recently, Copan launched PhenoMATRIX PLUS, a software designed to streamline workflows and better manage culture-related data. For culture-based diagnostic, AI algorithms (PhenoMATRIX), coupled to WASPLab, can assess colony growth, color, hemolysis, and morphology. Thus, AI algorithms can sort and proceed to predefined follow-up operations in each culture media plate. The performance of these AI algorithms was evaluated in various studies (11 – 16).

The objectives of the present study were: (i) to assess the performance of PhenoMATRIX and PhenoMATRIX PLUS for the screening of MRSA from nasal and inguinal/perineal ESwabs using chromogenic media as compared to the manual reading of culture images and (ii) to evaluate PhenoMATRIX PLUS for the automatic transmission of negative results to the Laboratory Information System (LIS) and for the automatic discharge of the negative plates from the incubators.

## MATERIALS AND METHODS

### Setting

This study was conducted at Geneva University Hospitals, a Swiss tertiary care center. The bacteriology laboratory processed 197,120 clinical specimens in 2022 including 35,276 screening for multidrug-resistant organism carriers. Current hours of operation of the laboratory span from 7:30 a.m. to 7:30 p.m. (7/7). All media plates are imaged at predefined time points each day of incubation on the WASPLab, but the identification of pathogens and their AST is only performed during the day shift (from 8:00 a.m. to 4:30 p.m.).

### Manual work-up for MRSA

Nasal and inguinal/perineal MRSA screening ESwabs (490CE.A, Copan Italia S.p.A. Brescia, Italy) were streaked on CHROMID MRSA plates (bioMérieux, Marcy l'Etoile, France) by the WASP and incubated in the WASPLab. Several high-resolution digital images were taken under different light and exposure conditions at two incubation time points (0 h and 18 h). The final incubation time point was validated previously (17). The digital images were manually read by the technologists. For all the presumptively positive MRSA colonies according to their color (green) on CHROMID MRSA plate, a qPCR assay targeting *femA* and *mecA* was performed using Thermo Fisher Scientific—QuantStudio 5 Real-Time PCR and Absolute qPCR MasterMIX Thermo Scientific ABgene according to the protocol published by Francois et al. (18). In addition, AST was carried out by disk diffusion on all *femA* positive isolates.

### PhenoMATRIX: machine learning phase

We collected culture plate images of 6,771 non-duplicate specimens classified by manual reading as MRSA positive or MRSA negative. These images were used as ground truth to train the convolutional neural networks (i.e., a machine learning approach).

### PhenoMATRIX: validation phase

The validation of the settings defined during the learning phase was performed on 17,223 non-duplicate specimens sequentially referred to our laboratory between 09 February 2022 and 08 May 2023. Digital images of the chromogenic media plates were prospectively analyzed by PhenoMATRIX. To ensure maximal consistency, the same plate images were read manually by the technologists, but they were kept blind of the results provided by PhenoMATRIX. For each discrepancy between manual reading and PhenoMATRIX results, the corresponding culture images were reviewed by clinical microbiologists.

We examined for the 17,223 specimens the difference in TAT between WASPLab and the manual reading according to the time when the culture images were available on the WASPLab (i.e., after 18 h of incubation).

### PhenoMATRIX PLUS: evaluation of the performances

**Fig 1:**
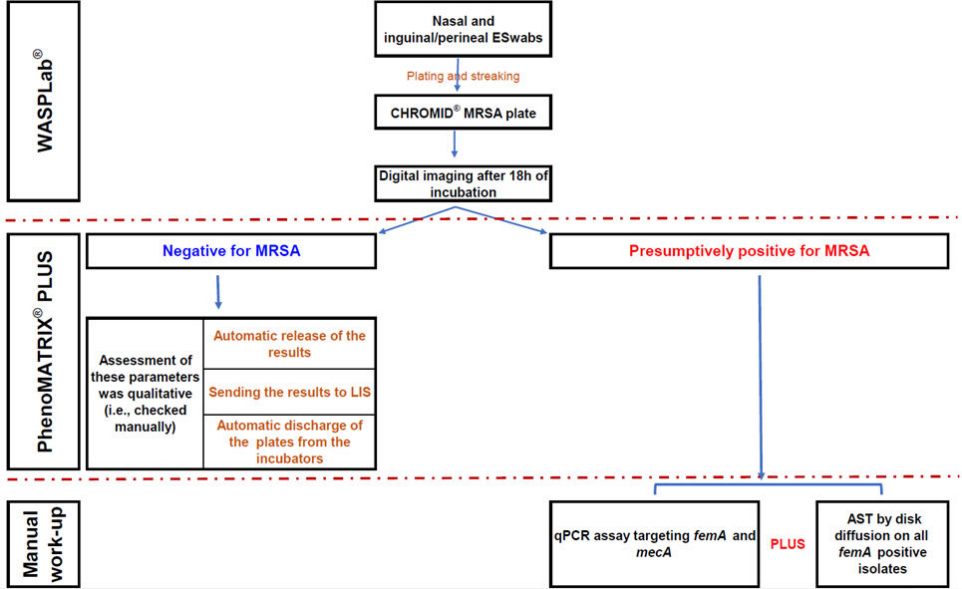
Algorithm used to assess the performance of PhenoMATRIX PLUS.

PhenoMATRIX PLUS was assessed against the manual reading on 1,409 specimens sequentially referred to our laboratory between 23 May 2023 and 03 July 2023. The automatic release of the negative results to the LIS and the automatic discharge of the negative plates from the incubators were checked manually by the staff for all the 1,297 MRSA-negative specimens. Figure1 shows the algorithm used for this evaluation.

## RESULTS

### PhenoMATRIX performance for the detection of MRSA

**Fig 2:**
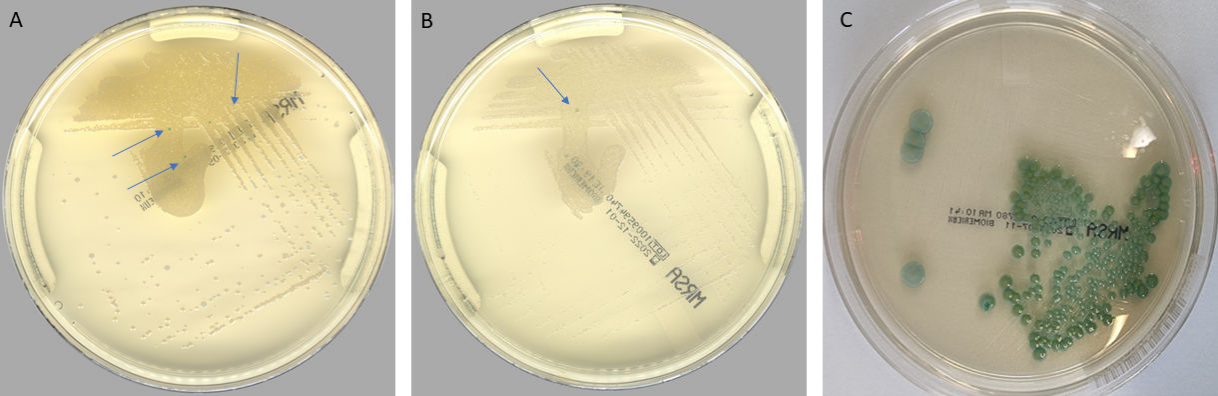
Digital images of the two PhenoMATRIX MRSA false negative results. (A and B) MRSA false negative: small and non-specific colonies (blue arrows) growing on CHROMID MRSA agar. These colonies were not detected as MRSA positive by PhenoMATRIX. (C) A representative digital image of CHROMID MRSA agar growing MRSA.

Table 1 depicts the clinical performance of PhenoMATRIX for the detection of MRSA compared to the manual work-up results. Among the 17,223 specimens analyzed in the validation phase, the MRSA positivity rate was 5% (868/17,223). According to the results provided by the manual work-up, the sensitivity and the specificity of the PhenoMATRIX were 99.9% [95% confidence interval (CI), 99.2%–99.9%] and 99.1% (95% CI, 99.0%–99.2%), respectively. Figure 2 depicts the culture images of the two false negative cases reported by PhenoMATRIX. We noted the presence of few small colonies with pale colors and poorly specific features for MRSA colonies. In contrast, these two cases were reported positive by the technologists and the molecular assays and AST confirmed the presence of MRSA.

**TABLE 1:** Performance of PhenoMATRIX for the detection of MRSA from nasal and inguinal/perineal screening ESwabs on chromogenic media compared to manual reading

		Manual work-up		
		Negative	Positive	Total
PhenoMATRIX	Negative	16,209	2	16,211
	Positive	146	866	1,012
	Total	16,355	868	17,223

**Fig 3:**
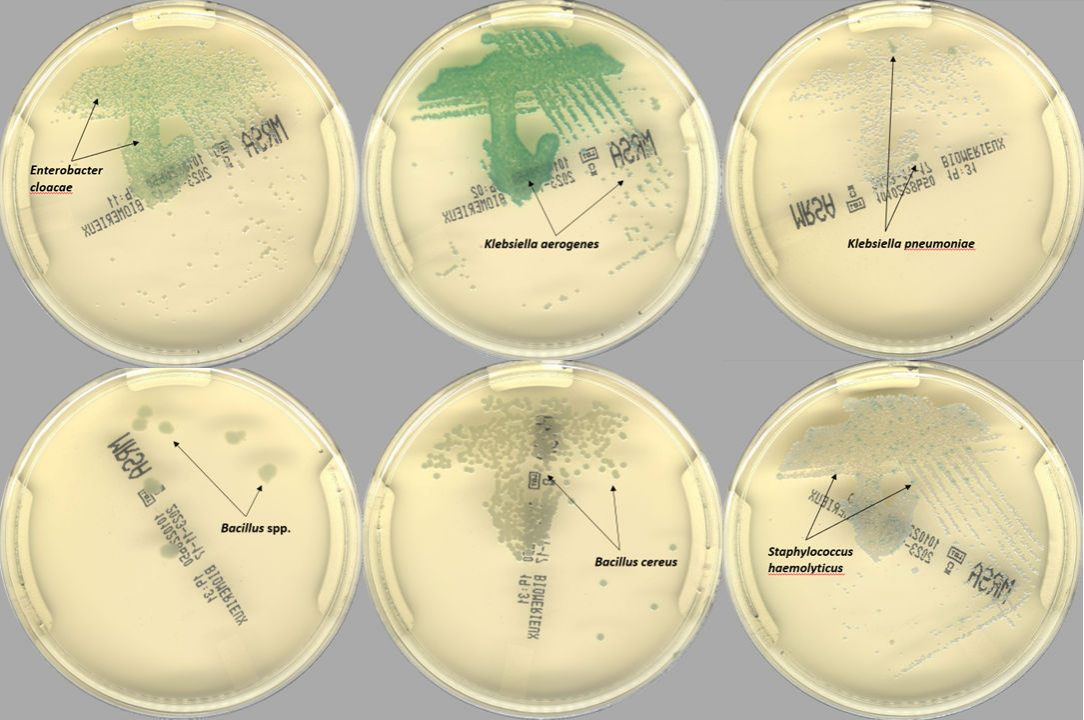
Representative digital examples of the most frequent PhenoMATRIX MRSA false positive results.

Among the 17,223 specimens analyzed by PhenoMATRIX, 146 were determined as false positive. All such cases were reviewed by clinical microbiologists, and the absence of MRSA was confirmed. Figure 3 depicts the culture images of some false positive cases. It may be noted that mixed growth of various species of Enterobacterales, coagulase*-*negative staphylococci, and *Bacillus* sp. constituted the most frequent false positive cases (about 85%), followed by non-specific colors on chromogenic plates. When compared to the large number of cases included in the validation set, the rate of the false positive remained very low (0.85%, 146/17,223).

We did not observe any discordance between the qPCR assay targeting *mecA* and the AST (cefoxitin) results. Thus, non-*mecA* gene mediated methicillin resistance was not detected in this study.

### PhenoMATRIX PLUS for MRSA screening and culture plate processing

**Fig 4:**
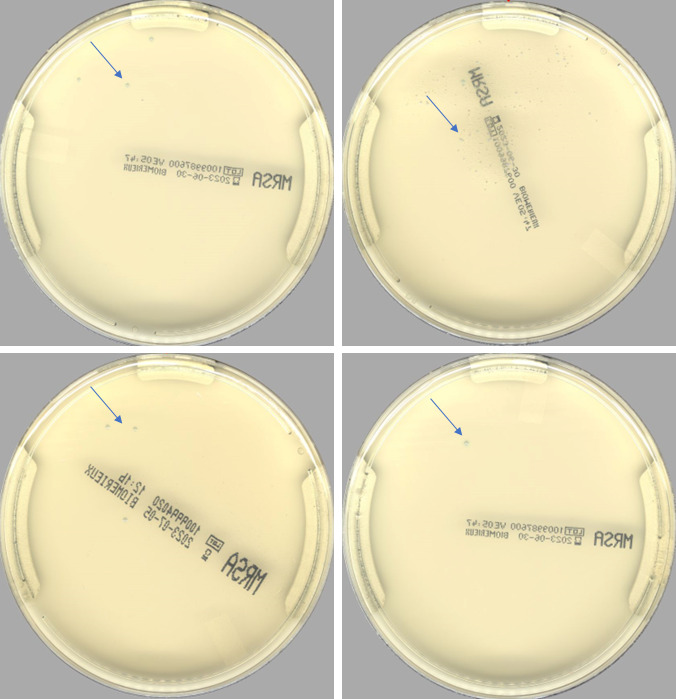
Digital images of four challenging cases that PhenoMATRIX PLUS classified as presumptively positive (blue arrows). MRSA was confirmed by molecular assays and AST.

**Fig 5:**
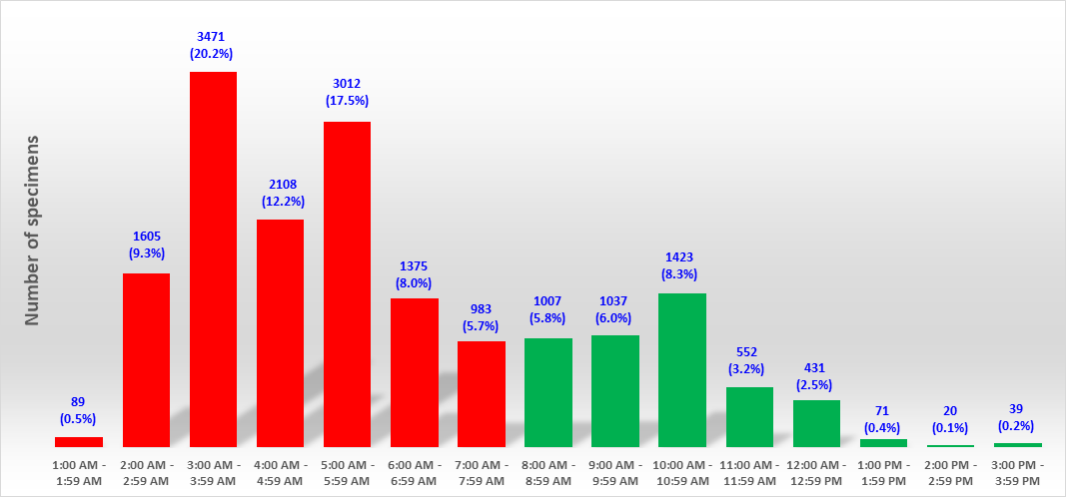
Times when the digital images of the chromogenic media were available on WASPLab after 18 h of incubation. Green bars correspond to specimens received during the hours of operation of the laboratory. Red bars depict off-shift hours.

**Fig 6:**
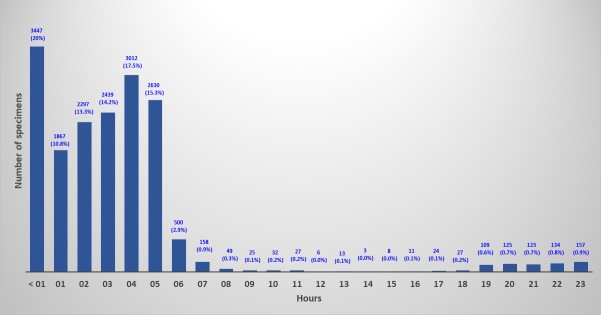
Difference in TAT between WASPLab and manual reading according to the time when the digital images were available on the WASPLab (i.e., after 18 h of incubation).

From 23 May 2023 through 03 July 2023, PhenoMATRIX PLUS analyzed 1,409 specimens. We manually reviewed the culture images of all these 1,409 specimens. No false negative result was observed (Table 2). Figure 4 shows the digital images of two challenging cases that PhenoMATRIX PLUS classified as presumptively positive and where MRSA was confirmed by molecular assays and AST. According to the manual reading of the culture images, the sensitivity and the specificity of PhenoMATRIX PLUS were 100% (95% CI, 92.1%–100%) and 95.2% (95% CI, 93.8%–96.1%), respectively. The MRSA positivity rate was 3.2% (45/1,409). The MRSA false positivity rate was 4.8% (67/1,409). The media plates of these 67 false positive cases were always sent automatically to the reading folder of the WASPLab so that they could be evaluated by the technologists. To provide a more accurate assessment of the potential impact of the automatic release of MRSA-negative results, and of the automatic discharge of the negative plates by PhenoMATRIX PLUS, we investigated for the 17,223 specimens used to validate the PhenoMATRIX when the culture images were available on the WASPLab, i.e., after 18 h of incubation. For 73% (12,643/17,223), the culture images were available between 1:00 a.m. and 7:59 a.m. For the remaining 27%, they were available during the opening hours of our laboratory (Fig. 5). Additionally, we defined the time elapsed between availability of such images and the manual release of such results to the LIS by the technologists. For 20% (3,447/17,223), the results were delivered to the LIS within 1 h following the availability of the culture images. In contrast, for 71% (12,245/17,223), the results were delivered to the LIS between 1 h and 5 h later. For the remaining cases (9%, 1,531/17,223), the results were delivered to the LIS between 5 h and 23 h later (Fig. 6). The TAT difference observed between the availability of culture images on the WASPLab and the manual reading is related to several parameters: (i) the confirmation assays of the presumptively positive cases, (ii) the hours of operation of the laboratory, and (iii) the laboratory workload, because in our workflow, the same technologist processes all screening cultures for resistant organisms including vancomycin-resistant *Enterococcus* (VRE), extended-spectrum beta-lactamases, and carbapenemase-producing Gram-negative bacilli.

**TABLE 2:** Performance of PhenoMATRIX PLUS for the detection of MRSA from nasal and inguinal/perineal screening ESwabs on chromogenic media compared to manual reading

		Manual work-up		
		Negative	Positive	Total
PhenoMATRIX PLUS	Negative	1,297	0	1,297
	Positive	67	45	112
	Total	1,364	45	1,409

**Fig 7:**
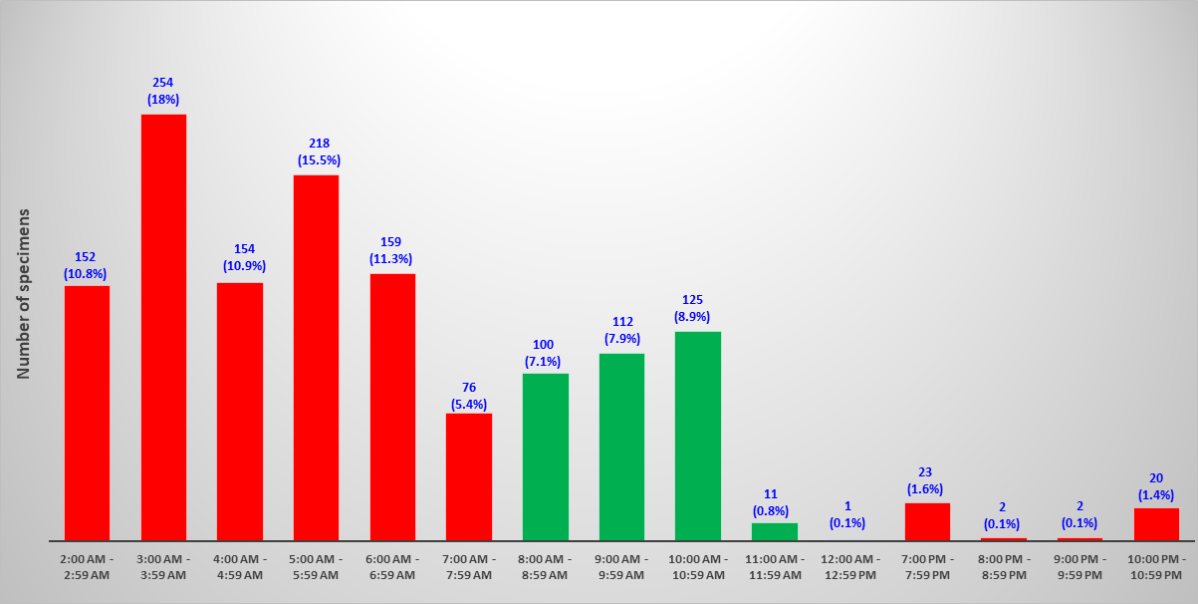
Times when PhenoMATRIX PLUS analyzed the MRSA culture images. Green bars correspond to specimens received during the hours of operation of the laboratory. Red bars depict off-shift hours.

Regarding the 1,409 specimens analyzed by PhenoMATRIX PLUS, 72% (1,013/1,409) of the culture images were available on the WASPLab between 2:00 a.m. and 7:59 a.m. (Fig. 7). Thus, all such negative results were delivered by PhenoMATRIX PLUS to the LIS before 8:00 a.m.

Overall, PhenoMATRIX PLUS automatically released 1,297 negative specimens, sent these negative results to the LIS, and automatically discarded all corresponding negative plates to the waste bin with a 100% accuracy.

## DISCUSSION

PhenoMATRIX software allows segregating negative from positive culture images based on the colony color profiles on chromogenic agar. The accuracy of such software has been established for MRSA (15), VRE (16), group A *Streptococcus* (13)*,* and group B *Streptococcus* (GBS) (11). In the study conducted by Faron et al., 57,690 anterior nares, throat, perineum, or wounds ESwabs were analyzed in several laboratories for the detection of MRSA. The overall prevalence of MRSA was 2.4%. The PhenoMATRIX software detected all plates that were considered positive by manual reading. More importantly, the software was able to detect 153 additional MRSA-positive specimens that were classified as negative by manual reading (15).

In the present study, we demonstrated that the performance of PhenoMATRIX PLUS for the detection of MRSA from nasal and inguinal/perineal swabs using chromogenic media was comparable to that of manual reading in terms of sensitivity and specificity. PhenoMATRIX PLUS ensured full management of the negative specimens from the analysis of the culture images to the delivery of the results to the LIS. This resulted in a significant reduction in workload since the technologists can now concentrate their efforts on the confirmatory tests of the presumptively positive plates. The negative and positive predictive values of PhenoMATRIX PLUS for the detection of MRSA from nasal and inguinal/perineal swabs using chromogenic media were 100% and 40%, respectively. We implemented therefore only the notification of negative results to the LIS, waiting for the results of additional tests to confirm (or not) the presumptively positive results.

In our previous study, we showed that the median TAT for negative reports decreased by almost half for MRSA screening specimens from 50.7 to 26.3 h (*P* < 0.001) with WASPLab (10). Using PhenoMATRIX PLUS coupled to WASPLab, negative results were immediately and automatically sent to the LIS around the clock, as this task was set a high priority. Negative plates were discarded from the incubators depending on the availability of the WASPLab (i.e., within less than 1 h), since that task was set a low priority.

**Fig 8:**
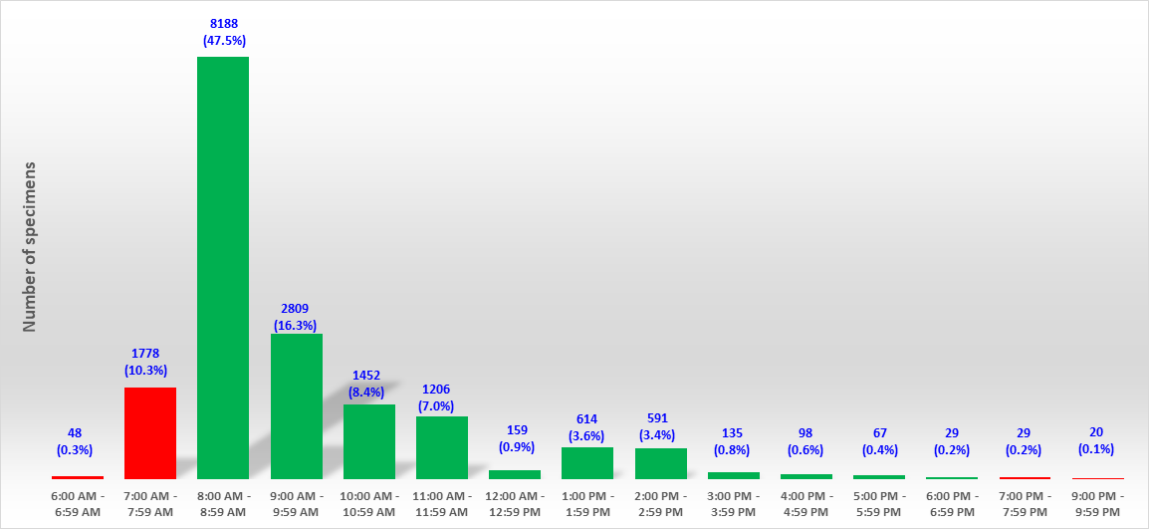
Delivery time of the 17,223 specimens included in the validation phase of PhenoMATRIX. Green bars correspond to specimens received during the hours of operation of the lab. Red bars depict off-shift hours.

In our current workflow, once negative MRSA results are automatically sent to the LIS by PhenoMATRIX PLUS, another software (Valab expert system, Flourens, France) automatically validates these results and updates the electronic patient record without any delay. The availability of the culture images on the WASPLab is very much tied to the time of arrival of the specimens at the laboratory and their subsequent processing. Figure 8 shows the delivery time of the 17,223 specimens included in the validation phase. The peak of activity was observed between 8:00 a.. and 9:00 a.m. (47%, 8,188/17,223). The WASPLab line implemented in our laboratory consists of two WASPs and three incubators. One WASP is dedicated to process the screening swabs for multidrug-resistant organisms. The specimens are streaked on chromogenic media and incubated on the WASPLab within a time interval of 5–120 min from the time of arrival of the specimens in the laboratory depending on the availability of the WASPLab. This time interval is be explained by the larger volumes of routine samples that create bottlenecks at certain times of the day, which impose establishing priority criteria based on specimen types.

The most obvious advantage of PhenoMATRIX PLUS is to save technologists' time on negative plates (i.e., >95% of the plates for MRSA screening in our institution). In addition to alleviating technologists' workload on a significant fraction of samples, it enables executing more rapidly other tasks in the laboratory. This helps mitigating the issue of shortage in qualified personnel. Specific studies assessing the precise impact on personnel load and on productivity are required. Finally, downstream effects in the clinical wards deserve also further studies (e.g., eliminating unnecessary infection control measures in the absence of drug-resistant organism).

### Conclusions

The performance of the PhenoMATRIX PLUS for MRSA highlights that this software can drastically decrease the time spent on negative plates with high accuracy, reducing the time to results independently of the opening hours of the laboratory. This allows additional flexibility for the management of the workflow and of the workload. Moreover, this AI software enables the continuous removal of negative media plates from the incubators, ensuring adequate management of the incubators' capacity. The scope of application of such software is very broad, as it could be implemented, after thorough validation, on a broad panel of culture-based analyses, such as urines, GBS screening, and other infection control screening specimens. Additional studies assessing patient outcomes, impact on antimicrobial prescriptions, and cost-savings assessments will further substantiate the integration of that software in microbiology laboratories.

## References

[1] Lee AS , Huttner BD , Catho G , Harbarth S . 2021. Methicillin-resistant Staphylococcus aureus: an update on prevention and control in acute care settings. Infect Dis Clin North Am 35:931–952. doi:10.1016/j.idc.2021.07.001 34752226

[2] Abbas M , Rossel A , de Kraker MEA , von Dach E , Marti C , Emonet S , Harbarth S , Kaiser L , Uçkay I . 2020. Association between treatment duration and mortality or relapse in adult patients with Staphylococcus aureus bacteraemia: a retrospective cohort study. Clin Microbiol Infect 26:626–631. doi:10.1016/j.cmi.2019.07.019 31357013

[3] Kluytmans J , Harbarth S . 2020. MRSA transmission in the community: emerging from under the radar. Lancet Infect Dis 20:147–149. doi:10.1016/S1473-3099(19)30539-0 31784370

[4] Lee AS , de Lencastre H , Garau J , Kluytmans J , Malhotra-Kumar S , Peschel A , Harbarth S . 2018. Methicillin-resistant Staphylococcus aureus. Nat Rev Dis Primers 4:18033. doi:10.1038/nrdp.2018.33 29849094

[5] Cherkaoui A , Schrenzel J . 2022. Total laboratory automation for rapid detection and identification of microorganisms and their antimicrobial resistance profiles. Front Cell Infect Microbiol 12:807668. doi:10.3389/fcimb.2022.807668 35186794 PMC8851030

[6] Thomson RB , McElvania E . 2019. Total laboratory automation: what is gained, what is lost, and who can afford it? Clin Lab Med 39:371–389. doi:10.1016/j.cll.2019.05.002 31383263

[7] Antonios K , Croxatto A , Culbreath K . 2021. Current state of laboratory automation in clinical microbiology laboratory. Clin Chem 68:99–114. doi:10.1093/clinchem/hvab242 34969105

[8] Kim K , Lee SG , Kim TH , Lee SG . 2022. Economic evaluation of total laboratory automation in the clinical laboratory of a tertiary care hospital. Ann Lab Med 42:89–95. doi:10.3343/alm.2022.42.1.89 34374353 PMC8368223

[9] Cherkaoui A , Schorderet D , Azam N , Crudeli L , Fernandez J , Renzi G , Fischer A , Schrenzel J . 2022. Fully automated EUCAST rapid antimicrobial susceptibility testing (RAST) from positive blood cultures: diagnostic accuracy and implementation. J Clin Microbiol 60:e0089822. doi:10.1128/jcm.00898-22 36173195 PMC9580353

[10] Cherkaoui A , Renzi G , Martischang R , Harbarth S , Vuilleumier N , Schrenzel J . 2020. Impact of total laboratory automation on turnaround times for urine cultures and screening specimens for MRSA, ESBL, and VRE carriage: retrospective comparison with manual workflow. Front Cell Infect Microbiol 10:552122. doi:10.3389/fcimb.2020.552122 33194794 PMC7664309

[11] Baker J , Timm K , Faron M , Ledeboer N , Culbreath K . 2020. Digital image analysis for the detection of group B Streptococcus from ChromiD strepto B medium using PhenoMatrix algorithms. J Clin Microbiol 59:e01902-19. doi:10.1128/JCM.01902-19 33087433 PMC7771474

[12] Foschi C , Turello G , Lazzarotto T , Ambretti S . 2021. Performance of PhenoMatrix for the detection of group B Streptococcus from recto-vaginal swabs. Diagn Microbiol Infect Dis 101:115427. doi:10.1016/j.diagmicrobio.2021.115427 34120035

[13] Van TT , Mata K , Dien Bard J . 2019. Automated detection of Streptococcus pyogenes pharyngitis by use of colorex Strep A CHROMagar and WASPLab artificial intelligence chromogenic detection module software. J Clin Microbiol 57:e00811-19. doi:10.1128/JCM.00811-19 31434725 PMC6812993

[14] Dauwalder O , Michel A , Eymard C , Santos K , Chanel L , Luzzati A , Roy-Azcora P , Sauzon JF , Guillaumont M , Girardo P , Fuhrmann C , Lina G , Laurent F , Vandenesch F , Sobas C . 2021. Use of artificial intelligence for tailored routine urine analyses. Clin Microbiol Infect 27:1168. doi:10.1016/j.cmi.2020.09.056 33038526

[15] Faron ML , Buchan BW , Vismara C , Lacchini C , Bielli A , Gesu G , Liebregts T , van Bree A , Jansz A , Soucy G , Korver J , Ledeboer NA . 2016. Automated scoring of chromogenic media for detection of methicillin-resistant Staphylococcus aureus by use of WASPLab image analysis software. J Clin Microbiol 54:620–624. doi:10.1128/JCM.02778-15 26719443 PMC4767952

[16] Faron ML , Buchan BW , Coon C , Liebregts T , van Bree A , Jansz AR , Soucy G , Korver J , Ledeboer NA . 2016. Automatic digital analysis of chromogenic media for vancomycin-resistant-enterococcus screens using copan WASPLab. J Clin Microbiol 54:2464–2469. doi:10.1128/JCM.01040-16 27413193 PMC5035414

[17] Cherkaoui A , Renzi G , Vuilleumier N , Schrenzel J . 2019. Copan WASPLab automation significantly reduces incubation times and allows earlier culture readings. Clin Microbiol Infect 25:1430. doi:10.1016/j.cmi.2019.04.001 30986560

[18] Francois P , Pittet D , Bento M , Pepey B , Vaudaux P , Lew D , Schrenzel J . 2003. Rapid detection of methicillin-resistant Staphylococcus aureus directly from sterile or nonsterile clinical samples by a new molecular assay. J Clin Microbiol 41:254–260. doi:10.1128/JCM.41.1.254-260.2003 12517857 PMC149566

